# Trigeminal Neuralgia and Its Surgical Treatment

**Published:** 1907-11-16

**Authors:** 


### Points in Surgery.

### TRIGEMINAL NEURALGIA AND ITS SURGICAL TREATMENT.

Trigeminal neuralgia is an affection which is characterised by the occurrence of paroxysms of pain in the area of distribution of the fifth cranial nerve. The age or sex of the patient does not seem to have any relation to the frequency of the complaint, although some authorities state that it is most commonly met with in middle-aged men. The pain, which is appallingly severe, comes on in paroxysms. At first the attack lasts only for a short time—sometimes as short as a few seconds—and there is an interval of days, or even weeks, before a second one supervenes; but as the disease progresses the paroxysms become prolonged and the intervals between them become shortened, so that the patient has little or no freedom from his agony, and eventually his life becomes a burden to him. The attacks seem in any given case to start in some branch of the fifth nerve which is constant, most commonly the maxillary division. The pain, however, spreads from that division to adjacent areas, and in advanced cases not only the whole distribution of the fifth nerve may be picked out, but the nerves supplying the adjacent areas of the neck and face may become involved. During the attack the patient presents a picture of extreme misery. The muscles of his face, and sometimes of his neck also, are subject to involuntary spasms, and he is constantly shifting his head into some new position in the hope that this may bring him ease. Counter-irritation seems to afford some relief, and to this end he presses on and rubs the affected area with his hand. But when the paroxysm has passed away the opposite is the case, and now the slightest stimulus appears sufficient to bring on another bout of pain. Eating, or even talking, may be sufficient to do this, and he therefore keeps himself absolutely still. On examination it will be found that certain localised areas are tender on pressure, and that these correspond generally to the points of emergence of the branches of the fifth nerve from their bony foramina.

### Two Distinct Classes.

Cases of trigeminal neuralgia may be divided into two distinct classes: (i.) Those in which some local focus of irritation can be found, and (ii.) those in which no organic cause can be detected. In the first class there can be no question that dental caries is the most common of such foci, particularly caries of one of the upper molars. In one recorded case the neuralgia was caused, not apparently by caries, but by the pressure of a metal stopping, which was separated from the nerve pulp by only a thin shale of bone. The medical man may be misled into overlooking dental caries by the fact that the pain is often referred at first to some tooth other than the carious one. Other causes which may lead to trigeminal neuralgia are abnormal conditions of the nose and of the eye. Among the former, rhinitis, nasal polypi, and suppuration in one of the accessory sinuses must be looked for; while, among ophthalmic causes, uncorrected errors of refraction account for a small percentage of the cases. But there is, as has been said, a second class of case, in which no such cause can be found, and there is no diseased condition of the nerve itself or of its nuclei to account for the symptoms. In the days when the Gasserian ganglion was first removed for this complaint it was thought that a microscopical examination of the tissue removed would demonstrate the presence of an inflammatory change in the trunks of the nerve or its nuclei. This is not, however, the case. In nearly all cases the ganglia which are removed are found to appear normal under the microscope. The cases in which some organic cause can be found are more easy to treat, for if the local focus be dealt with by suitable measures, as, for instance, the removal of a carious tooth or a nasal polypus, or the correction of any errors of refraction, the neuralgia tends to disappear. But in the primary, or so-called idiopathic cases, in which no apparent cause can be found, the disease tends to progress steadily in spite of medical treatment. What usually happens is that the patient rings the changes on the more ordinary antispasmodic drugs, such as phenacetin, antipyrine, and antifebrin, until he finds which one of them gives him most relief, and he takes increasing doses of this, until it loses its power to allay his pain; and finally he finds that the only drug that gives him any real comfort is morphia.

### Surgical Treatment.

The following methods of surgical interference have been devised from time to time in the hope of giving relief in these distressing cases:—(i.) Supraorbital neurectomy. The nerve trunk is exposed through a small incision in the line of the eyebrow as it emerges through the supraorbital notch. It is pulled up, and as much as possible of the nerve trunk is removed. The disadvantage of the operation is that the neuralgia is rarely, if ever, confined to the supraorbital division, and, even if it is, the relief afforded by a simple neurectomy is not likely to be permanent.

(ii.) Infraorbital neurectomy. A short incision is made along the natural fold of the cheek over the infraorbital foramen. The nerve is seized as it emerges and pulled out as far as possible. In most cases no improvement results, because the dental branches are not removed by this procedure. To overcome this objection a more radical operation was devised. A V-shaped incision is made in the skin of the cheek, and a portion of the anterior wall of the antrum of Highmore is taken away with a chisel. The floor of the infraorbital foramen is removed, and the infraorbital nerve can be torn out entire from its attachment posteriorly.

(iii.) Neurectomy of the inferior dental nerve. This is done by making an incision in the mucous membrane of the mouth opposite the inferior dental spine. The internal lateral ligament of the jaw, which is attached to this spine, is divided with scissors. The nerve can be felt behind this, and can be brought into the wound with an aneurysm needle, and the exposed portion can then be cut away.

None of the foregoing operations are satisfactory, because, firstly, the pain is rarely confined to any single division of the nerve, and, secondly, however thoroughly the operation is done, the relief afforded appears to be only temporary.

### Operations for the Removal of the Gasserian Ganglion.

It was not until the operation for removal of the Gasserian ganglion was devised that any genuinely successful form of treatment for this condition was available to the surgeon. The credit for having first suggested this operation belongs to Mr. Rose. In 1892 he published records of a series of cases in which he had successfully removed the Gasserian ganglion by exposing the base of the skull and trephining it in the neighbourhood of the foramen ovale. The operation itself is a long and complicated one, and has been almost entirely superseded by the method to which the name of Hartley-Krause has been given, so that its steps will not be described in detail.

The Hartley-Krause operation for removal of the Gasserian ganglion is necessarily a severe one, and is associated with some risk. But there is no doubt that in advanced cases of trigeminal neuralgia its performance is justifiable, because the relief afforded is immediate and permanent. It should be advocated in those cases in which the neuralgia has resisted all known forms of medical treatment and is increasing in severity, so that the patient's life is literally a burden to him. Of course the dangers of the operation must be explained to the patient; but it will generally be found that he is prepared to take any reasonable risk rather than to continue his present miserable existence.

### Technique of the Hartley-Krause Operation.

Before the operation the patient's head should be completely shaved. It is not sufficient to remove the hair only on the affected side, because the remaining hair cannot be made aseptic; this is very essential, since, if this operation is not aseptically performed, death from meningitis is the most probable result. The operation is performed as follows:—An incision in the scalp is made convex upwards, extending from the external angular process in front to the tragus behind, and reaching as high as the supratemporal crest. This flap is turned down with all the structures down to the bone. The temporal crest is then trephined with a large trephine. This must be done very carefully, because it is extremely important not to injure the dura mater; and it must be remembered that the skull in this region is very uneven, being thin in some places and thick in others. When the circle of bone has been removed, the opening in the bone is enlarged with a rongeur until it is the same size as the skin wound. The dura mater must now be gently freed from its attachment to the base of the skull with a blunt dissector. In some cases this can be done quite easily, but in others the moment any attempt is made to elevate the dura the wound is filled with blood, which oozes from the veins on the surface of the membrane. This hæmorrhage is never sufficient to cause any danger to the patient, but it is excessively annoying to the surgeon, because it allows him only temporary glimpses of the field of operation. Sometimes the welling up of blood is so persistent that it is better to close the wound and allow a week to elapse before making another attempt, in the hope that some of the torn venous radicles will have closed in the interim. It is no exaggeration to say that the difficulty of the operation is directly proportional to the amount of venous oozing which is encountered. As the dura mater is gradually freed the brain is held out of the way on a broad, flat, metal retractor. The first structure which is met is the trunk of the middle meningeal artery as it comes through the foramen spinosum. This must be divided between ligatures. The ligatures must be applied and tied with two pairs of specially long dissecting forceps. A little deeper down the second and third divisions of the fifth nerve are met at the foramen rotundum and ovale respectively.

The dura mater is then carefully stripped up from the upper surface of the ganglion. Much discussion has arisen as to whether the whole ganglion and all three divisions of the fifth nerve should be removed, or only the outer two-thirds with the second and third divisions. There is much to be said in favour of the latter. The neuralgia rarely affects the ophthalmic division with the same severity as the maxillary and inferior dental branches. Moreover, removal of the ophthalmic division is apt to lead to keratitis, on account of the subsequent insensibility of the cornea; and, again, its relation to the oculomotor nerves as they lie in the wall of the cavernous sinus is so intimate that these are not infrequently damaged at the operation, so that ptosis or strabismus may result. In an ordinary case, therefore, it is sufficient to divide the inferior dental and maxillary roots at the foramen ovale and rotundum respectively, and to remove them with the outer two-thirds of the Gasserian ganglion. Venous oozing is arrested with sponge pressure, and the external wound is closed.

### Results of Surgical Treatment.

The results of this operation are most gratifying. The pain disappears at once, and, as far as can be judged at present, recurrence is almost unknown. There is, of course, complete anæsthesia of that side of the face, and, if the motor division has been removed, atrophy of the corresponding muscles of mastication; but in spite of this the patients appear to be able to manage their food quite well with the muscles of the other side.

The operation is, of course, a severe one, and is associated with a definite mortality from shock and sepsis; but when one considers the intolerable condition of a patient with advanced epileptiform neuralgia there can be no doubt that, as Mr. Jonathan Hutchinson, jun., says, the introduction of the Hartley-Krause method forms one of the most important surgical gains of the last twenty-five years.

